# An Integrated Framework to Conceptualize and Develop the Vancouver Airways Health Literacy Tool (VAHLT)

**DOI:** 10.3390/ijerph18168646

**Published:** 2021-08-16

**Authors:** Iraj Poureslami, Jacek Kopec, Noah Tregobov, Jessica Shum, Rick Sawatzky, Richard Hohn, J. Mark FitzGerald

**Affiliations:** 1 Faculty of Medicine, Respiratory Medicine Division, University of British Columbia, Vancouver, BC V5Z 1M9, Canada; iraj.poureslami@ubc.ca (I.P.); noahtregobov@gmail.com (N.T.); jessica.py.shum@gmail.com (J.S.); 2 School of Population and Public Health, University of British Columbia, Vancouver, BC V6T 1Z31Z3, Canada; jkopec@arthritisresearch.ca; 3 School of Nursing, Trinity Western University, Langley, BC V2Y 1Y11Y1, Canada; Rick.Sawatzky@twu.ca; 4 Department of Psychology, Simon Fraser University, Burnaby, BC V5A 1S61S6, Canada; richard_hohn@sfu.ca

**Keywords:** health literacy, conceptual framework, patient engagement, functional measurement tool, disease-management outcomes, asthma, COPD

## Abstract

There is currently no comprehensive tool to assess the functional health literacy (HL) skills of chronic airway disease (CAD) patients. The purpose of this article is to describe the development of a new HL measure, the Vancouver Airways Health Literacy Tool (VAHLT). The tool was developed through the following phases: (1) *Tool conceptualization*, consisting of: (A) a systematic review (SR), (B) focus group sessions with CAD patients to understand barriers and facilitators to CAD management, (C) a survey with key-informants to obtain strategies to mitigate self-management barriers and validate patient-derived topics, and (D) respiratory physicians’ review of the topics; (2) *Scenario and item development;* and (3) *Tool testing and content validation*. The SR identified the lack of a valid HL measurement tool for CAD patients. Patients provided an initial shortlist of disease-related self-care topics. Key-informants helped to finalize topics for inclusion. Respiratory physicians and patients contributed to the development of a scenario-based questionnaire, which was refined during three rounds of testing to develop a 44-item instrument comprising nine self-management passages. We highlight the holistic process of integrating information from the literature with knowledge gained from key stakeholders into our tool framework. Our approach to stakeholder engagement may be of interest to researchers developing similar tools, and could facilitate the development and testing of HL-based interventions to ultimately improve patient outcomes and reduce the burden on the healthcare system.

## 1. Introduction

Health literacy (HL) is defined by the Canadian Expert Panel on HL (CEPHL) [1] and Calgary Charter on HL (CCHL) [2] as having five core domains encompassing “an individual’s capability to access, understand, communicate, evaluate, and use health information and care services to make informed decisions for one’s health and well-being”. Numeracy is also considered to be a HL skill [3]; however, it is typically assessed across the HL domains, rather than as an independent entity [4]. In Canada, over 60% of the adult population (≥16 years of age) and 88% of seniors (>65 years of age) have insufficient abilities to manage their health or make informed health-related decisions [5,6,7]. Low HL is associated with poor health outcomes and excess healthcare spending in a variety of chronic health conditions [8,9], including asthma and COPD, the two main chronic airway diseases (CAD) [10,11,12,13,14]. HL challenges in CAD management are associated with unnecessary hospitalizations and emergency department visits [15,16], reduced medication adherence [17,18], lower quality of life [15,18], and increased mortality [16,17,18,19]. Inversely, improved HL is associated with better health outcomes and reduced health-related costs [20,21,22].

An aging population means increasing rates of chronic diseases [23,24,25]. Decision makers have recognized enhancing patients’ HL levels and healthcare professionals’ communication skills as important strategies to improve chronic disease management [26,27,28,29,30,31]. Shortcomings in existing HL measurement tools have hindered clinicians from identifying and modifying the health behaviours of those with low HL, and have prevented researchers from establishing the longitudinal effects of HL on outcomes [10,32,33]. The major criticisms of existing HL tools are that they: (1) have been developed for the general population and with little to no input from patients, limiting their validity and applicability to specific disease contexts [34,35,36,37]; (2) are brief screening-based tools used to identify a patient’s HL level that mainly assess reading, pronunciation, word recognition, and comprehension skills (general literacy) of medical terms and health information, and fail to assess HL domains and skills that are relevant to complex, real-world situations in healthcare settings [38,39]; (3) rely excessively on patients’ self-evaluation, i.e., one’s perceived ability to competently act within a hypothetical health-related scenario, as opposed to measuring one’s performance-based HL abilities, whereby patients display HL skills in a realistic situation [40]; and (4) are primarily developed for research purposes, and lack transferability to clinical practice [41,42].

To our knowledge, there is no published study that measures the performance-based HL skills of patients with CAD. Thus, a call to embrace HL as a significant influence in CAD management has been accompanied by the recognition of the need to measure HL comprehensively and accurately [10,14,17,19]. The objective of this article is to summarize the holistic, integrated approach (i.e., involving key sources and informants) we followed in developing our performance-based Vancouver Airways Health Literacy Tool (VAHLT). Conceptualization of the tool was grounded in a literature review, our previous work on the role of HL in chronic disease management, and consultations with patients and professionals on the perceived applicability and impact of HL on CAD outcomes.

Across the multiple conceptualization and development stages, our specific aims were: (1) to demonstrate the need for a performance-based HL measurement tool for CAD patients; (2) to develop a conceptual framework for our tool, combining the perspectives of patients with the expertise of health researchers and respiratory clinicians; (3) to develop scenarios and questions able to measure HL skills of CAD patients within the five core domains of HL and numeracy, pertaining to disease self-management practices; and (4) to verify that the tool has content validity—that is, the framework is comprehensive, and the selected topics, scenarios, and items are relevant, practical, and effective from both patients’ and professionals’ perspectives. It is important to note that some components of the VAHLT conceptualization and development process have been described previously [43,44,45,46,47,48,49,50,51]. Detailed results on psychometric and clinical validation studies will be addressed in future publications.

## 2. Materials and Methods

### 2.1. Theoretical Grounding and Rationale

A validity-driven approach [48] was employed in the development of the VAHLT. We applied the five-domain HL definition suggested by CCHL [2], which is widely used in the HL field [38].

**Figure 1:**
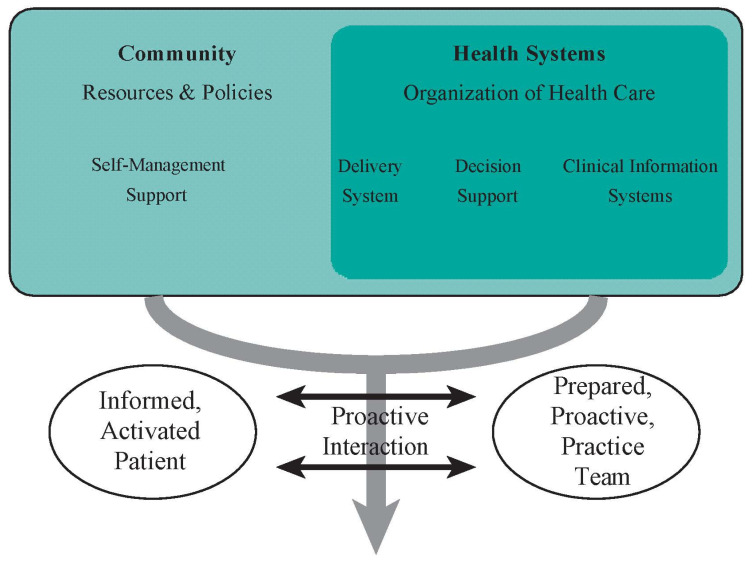
The Chronic Care Model (CCM)—community and professional engagement.

In 2008, the CEPHL recognized the need to improve HL measures, as well as empower patients to properly manage their disease [1,52]. In 2013, we convened a week-long international workshop to ensure that our research plan aligned with HL research priorities, while integrating patients’, healthcare professionals’, and researchers’ perspectives [53,54]. The concept of the Chronic Care Model (CCM) [55] (Figure 1) was applied as a framework to facilitate participants’ engagement in discussing HL and related disease-management topics. Attendees identified important aspects of social context and its influence on HL, including barriers to navigating the health system and actively engaging in self-management practices, challenges among ethno-cultural and marginalized communities, and the role of healthcare professionals and systems in addressing HL gaps between patient population groups. The CCM model was applied throughout our tool-development process. One critical outcome of this engagement was endorsement of the need for a performance-based HL tool to facilitate better CAD management. Our proposed initiative also aligned with the CEPHL’s emphasis on conceptualizing HL in chronic disease management and the changes in communication strategies, clinical practice, research agendas, and policy procedures required to improve Canadians’ HL [1,52]. Additionally, our initiative was enriched through knowledge gained during various qualitative and interventional HL and disease management studies with asthma and COPD patients [56,57,58,59,60]. This enabled us to identify patient challenges across HL domains, such as understanding verbal/written instructions and accessing information and services.

### 2.2. Study Procedures

**Figure 2:**
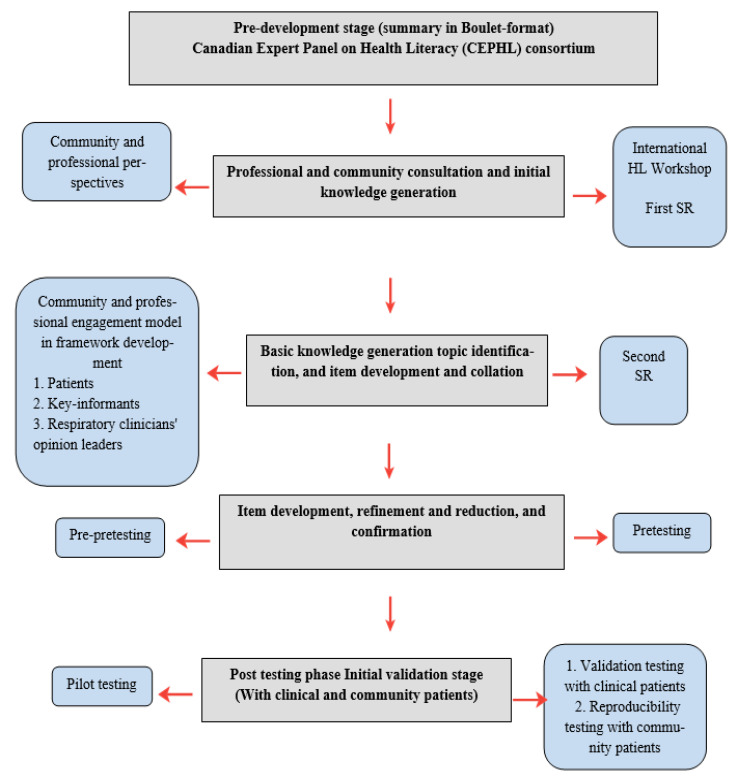
Conceptualization and development of the asthma and COPD disease management health literacy tool: needs assessment and pre-validation stages.

The tool framework development process comprised three interrelated phases of consultation with key-stakeholders, item identification and refinement, and testing. The steps undertaken during the study period (2015–2020) are outlined in the following sections and summarized in Figure 2.

#### 2.2.1. Phase I: Tool Conceptualization

**Figure 3:**
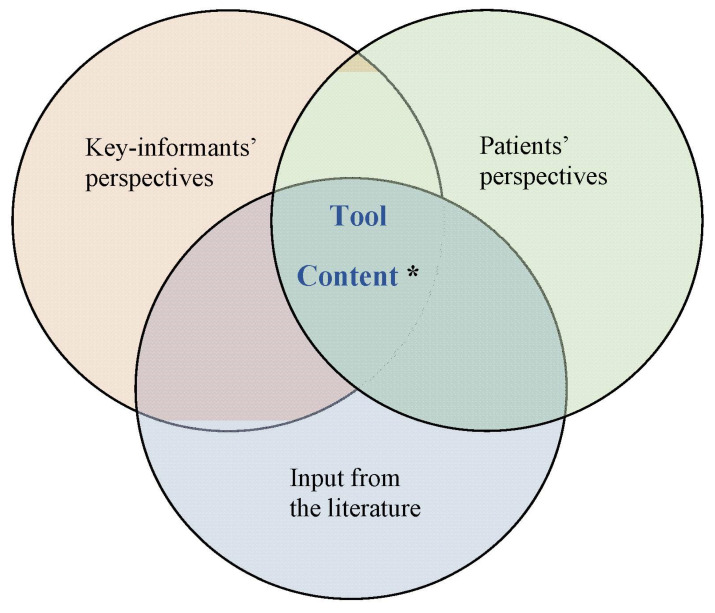
Three main sources for the VAHLT development for conceptualization: key-informants’ perspectives, patients’ perspectives, and input from the literature. The asterisk ‘*’ indicates *the common prespectives of patients and key-informants that matched with the literature were selected and applied in the development of our health literacy tool framework*.

The goal of this phase was to identify disease-management topics representing content areas for the tool. To ensure content relevance and comprehensiveness, we obtained input from the literature and engaged both patients and professionals (Figure 3).

We conducted an SR of measures used to evaluate the HL skills of patients with CAD. In parallel, we established an advisory panel (“knowledge hub”) that included patient partners, national and international HL experts, and respiratory specialists. Patients were recruited through clinical/network referrals and experts via snowball sampling [61] initiated by a focused search for multi-disciplinary scientists with expertise in HL and chronic disease management [62].

Next, we hosted focus group sessions with English- and French-speaking CAD patients from six collaborating sites across Canada. The goal of the group sessions was to understand patients’ perspectives on various aspects of CAD self-management. Participants were asked to talk about barriers and challenges pertaining to the five HL domains (including numeracy), as well as facilitators of optimal disease-management practices. They discussed disease-management topics, including their perceived importance, and provided questions for each topic that they would ask healthcare professionals. The study procedures, including focus-group structure and qualitative analysis, have been described in-depth in previous publications [43,45,47,49,50,62]. 

Subsequently, we conducted in-person or telephone/Skype interviews with national and international key-informants to review our framework and document their perspectives on our proposed process. We aimed to obtain professional insights about the role of HL in CAD management, elicit strategies to mitigate the challenges/barriers expressed by the focus-group participants, and verify the disease-management topics proposed by the patients. Following interviews, expert respiratory clinician–scientists from across Canada reviewed the patient-identified topics and shared optimal disease-management skills that a CAD patient should have to properly manage their condition. The data collected from patients and professionals were analyzed using NVivo software (QSR International, version 12) with coding via thematic analysis [63]. We have reported the process and findings of the informant interviews and respiratory experts’ input elsewhere [30,43,46,62]. Knowledge generated from the SR and consultations with patients and professionals allowed us to identify and verify important disease-management topics, and understand the internal stimuli (e.g., beliefs, worldviews) and external barriers (e.g., health system and socioeconomic factors) to engage in CAD self-management [48].

#### 2.2.2. Phase II: Scenario and Item Development (Preliminary Version Development)

Initially, we analyzed a list of potential factors (e.g., barriers and facilitators across the five core HL domains and numeracy) that were suggested by participants in Phase I. We then developed a preliminary set of scenarios for our measurement tool. Passages were presented in different formats (e.g., writing, pictures, diagrams, maps, website screenshots, charts, forecasts, etc.) and took different approaches (e.g., role playing/story-related format, writing answers in sentences, check-in, or pictorial and educational informative statements), just as one would encounter in a real-world healthcare setting. We then developed questions (items) for each scenario. The item bank was refined by assessing and eliminating repetitions, and integrating patients’ and professionals’ recommendations from the previous phase. Each item/question was structured in a multiple-choice format (four option answers) with only one correct answer. Next, the scenarios and corresponding questions were presented to selected patients and key-informants from the advisory panel to review the content, including each scenario and item’s perceived relevance to disease management (both patients and professionals) and HL domains (only HL researchers). We sought a consensus among patients and professionals on the confirmed topics and items to be included in the tool. Further revisions were applied to the tool’s format to ensure the inclusion of important disease-management topics and all HL domains and numeracy, and further elimination of unnecessary or repeated items.

#### 2.2.3. Phase III: Tool Testing and Content Validation

To confirm the content validity of the VAHLT, the revised tool was pre-tested in 2017 with a new cohort of CAD patients and selected key-informants to obtain a final consensus regarding the tool’s overall layout in terms of the scenarios, the order of topics, and comprehensiveness of the item pool to measure the core HL domains [49]. Patients completed the questionnaire in person and were instructed to write their comments and suggestions about the tool, including any difficulties in understanding the scenarios, as well as its perceived relevance to CAD management. The key-informants received a digital copy of the tool and were informed about the procedures to provide feedback (e.g., rating of items in terms of relevance to CAD disease management and the HL domains, and the difficulty level of the questions).

Finally, a professional adult patient educator conducted a thorough evaluation of the tool to ensure that the content was user-friendly, improved the layout and orientation of certain items and diagrams, and refined the wording to an appropriate reading level. The aim was to ensure that our proposed tool was capable of distinguishing differences that were attributable to HL skills as opposed to other factors, including prior disease-related knowledge, or complex terms and medical jargon. The results of this phase have been reported in separate publications [43,49,50].

### 2.3. Subsequent Testing of VAHLT 

Following Phases I, II, and III, we tested the VAHLT in two cohorts of CAD patients recruited from six collaborating sites across Canada to reduce the items and assess the measurement properties of the final tool. These studies will be reported in future publications.

### 2.4. Ethics

Study approval was obtained from the University of British Columbia Clinical Research Ethics Board (project approval number: H15-01954). Ethics approval was also received from the Institutional Review Boards at each of the participating centers prior to recruitment. Written informed consent was obtained from all subjects involved in the study.

## 3. Results

### 3.1. Phase I: Tool Conceptualization 

#### 3.1.1. Systematic Review

Our SR [33] identified major deficiencies in currently available screening-based HL instruments, and confirmed the need for a functional HL tool for CAD patients. The key findings pertaining to our tool development were: (1) the five core HL domains outlined by the CCHL [1,52] were underrepresented among the tools; (2) overreliance on the “understand” HL domain was common, indicating the potential misuse of word comprehension as a proxy for HL skills; and (3) use of a self-evaluative design as the dominant approach, meaning patients were assessed based on the indication of their perceived level of competence to act in a given health scenario, as opposed to actually completing the task whilst displaying their functional HL skills. Additionally, numeracy assessment was absent from most articles, and no HL measurement tool had been developed specifically to assess the functional HL skills of adult CAD patients. Our findings provided two main insights. First, the review identified the need to assess HL in a complete manner (accounting for all domains). Second, it highlighted the importance of having patients complete realistic health-related tasks to accurately gauge their HL capacities (rather than self-evaluate)—a fundamental aspect of our performance-based tool.

#### 3.1.2. Patient Involvement

The SR identified a lack of patient engagement in the development of most existing HL measurement tools. This is a significant limitation, with potentially serious implications for the validity of the resultant instruments [33,38,43,64,65]. During 16 focus-group sessions with CAD patients, participants mentioned challenges in accessing relevant information, difficulty understanding treatment instructions, limited ability and inadequate knowledge to act on information, and the constraints of daily demands in managing their disease. These data are discussed in detail elsewhere [46,62]. Patients’ responses were analyzed across the five core HL domains and included the following key insights into the design of our tool: (1) Access: participants suggested clarifying the different ways to access and obtain health information and services. They defined “passive access” as the availability of health information provided by healthcare professionals, while “active access” was identified as patients navigating and finding needed information independently. “Lack of access” was also identified as a major barrier to receiving needed health information and care services. (2) Understand: patients’ perception of understanding is often related to their difficulty in comprehending the complexity of the information, particularly treatment instructions (i.e., how to adhere), triggers of disease worsening (i.e., how to manage), and health education materials (i.e., how to apply). Medical terms/jargon used by healthcare professionals or found in written material contributed to patients’ inability to understand health information. Finally, the participants talked about challenges in grasping numerical aspects of their action plan, tests results, or instructions received from care providers that prevented them from fully comprehending and following the information. (3) Evaluate: healthcare professionals’ credibility made them the most trustworthy information source for many participants. Several provider-related factors were cited when patients evaluated the trustworthiness of the information received from a care provider: communication skills, confidence level, professional reputation, and specialization/credentials. When evaluating the validity of sources, participants mentioned that they judged information based on the source and whether they deemed it to be reputable and correct. (4) Communicate: descriptions of communications were often centered on improper or inadequate patient–provider interactions. Patients mentioned a lack of proper information exchange between a care provider and a patient, including managing an exacerbation and treatment side-effects. Similarly, challenges were identified in treatment adherence, as many patients noted that they did not always adhere to their prescribed treatments, often due to miscommunications with care providers. (5) Use/Apply: participants mentioned two main factors that they believed promote the use of health information: (1) that it was from a credible source, and (2) receiving the information from a care provider when they are ready to accept it in a suitable setting.

#### 3.1.3. Key-Informants Involvement

Forty-five key informants provided their perceived solutions to the barriers reported by patients. They noted that care providers should not only help patients to understand disease- and treatment-related information, but also to support their engagement in the self-management process by responding to their needs and concerns. They emphasized the provision of easy-to-follow instructions, increased information accessibility, and promotion of proper communication practices to enhance information uptake, all of which can enhance the patient’s confidence to apply the received information in making informed health decisions [66].

#### 3.1.4. Respiratory Clinician Validation

Seventeen respiratory physicians reviewed the disease-management topics and provided additional “actionable” items, which emphasized combining HL skills and self-management competencies for CAD patients to properly manage their disease [67]. The physicians agreed that to accurately measure HL, a patient should be placed in a situation consistent with their daily routine that would require them to act upon self-management information in a realistic scenario. This approach would allow us to measure performance-based skills, as opposed to a self-evaluation, and was consistent with findings from the SR [30] and suggestions made by key-informants [40,45,46].

### 3.2. Phase II: Scenario and Item Development (Preliminary Version Development)

Data from patients and key-informants helped us to identify, verify, and prioritize the most important disease-management topics relevant to both asthma and COPD patients. Participants frequently emphasized the need to enhance some scenarios with graphical and pictorial features, and also to integrate numeracy testing into the tool. It was noted that numeracy is a critical part of a patient’s decision-making process, as health and medical information is often presented as numbers, graphs, or tables [3,4,42,50,68]. In developing the VAHLT, we considered 159 potential disease-management elements suggested by patients and experts to develop a short list of the items by eliminating irrelevant and duplicated items. We recruited a further five asthma and five COPD patients in Vancouver, who had not previously participated in the study, to help develop the preliminary content of scenarios for each selected disease-management topic, as well as to suggest items pertaining to each topic from the item bank. Eventually, a draft tool was developed, that included 12 topics specific to asthma and COPD (eight shared + two asthma + two COPD) and 66 corresponding items (four to six items for each scenario) (Table 1). Each question tested one of the five core CCHL [2] domains and numeracy (Table 2). Appendix A contains a sample measurement-tool passage.

### 3.3. Phase III: Tool Testing and Content Validation 

We pre-tested the tool with patients and professionals (recruited from our knowledge hub) in two rounds. First, input from 19 CAD patients and 26 key-informants resulted in refinement of the scenarios and confirmed the inclusion of the selected topics and corresponding multiple-choice questions in our tool. Second, the revised tool was pre-tested with a new cohort of 75 CAD patients and 39 key-informants, who provided a final round of feedback regarding the tool’s relevance, difficulty, and practicability. The participants were positive about the relevance and appropriateness of the self-management topics (patients), layout of the scenarios (patients and key-informants), inclusion of all core HL domains and numeracy, and appropriateness of the multiple-choice scaling approach (patients and key-informants). Notably, participants deemed the scenarios to be informative and helpful in improving a patient’s understanding of disease-management practices. Based on the initial results, a tool consisting of 44 items and nine topics (seven shared + two disease-specific for asthma and COPD) was approved for a further psychometric validation (to be published elsewhere).

## 4. Discussion

We aimed to develop the VAHLT by combining patients’ perspectives with the viewpoints of professionals on key factors affecting CAD patients’ HL and self-management practices. Their involvement also helped to improve the VAHLT during multiple rounds of testing and feedback in terms of layout, content relevance, and applicability to CAD management. This article describes the process and framework we applied in the development of our HL measurement tool for CAD management.

During the development process, we identified several deficiencies in currently available HL tools. One such limitation was overreliance on self-evaluation and a patient’s perceptions of their HL skills, which are often inaccurate. In contrast, our performance-based tool was designed to measure HL abilities by asking participants to respond to questions based on information provided within each disease-management scenario. The aim of the VAHLT is to measure objective components of disease management, while minimizing potential biases associated with self-perceived or screening-based HL measures [38,42,52].

We found that patient involvement in prior HL tool-development processes was rare [33,48,49,51]. Our participants also mentioned, from their experience, that patients’ voices were often missing when developing educational interventions, particularly as related to disease-management practices [45,60,69]. In contrast to previously reported HL measurement tools [64,65,70,71], our community-engagement model [49,50,72] drew extensively on patients’ and health professionals’ insights to inform the process throughout its development. Evaluation of our community-engagement model supported its effectiveness [55,72], with implications for future initiatives in functional HL tool development. Patient participants were generally happy that their viewpoints were appreciated, and provided suggestions that were later integrated with those of healthcare professionals and HL researchers [43,62].

During our study, we identified numerous internal and external factors that prevent proper access to and use of health information. Our study participants indicated that for some Canadian population groups, including those with limited English and/or French language proficiency, low levels of general literacy, and living in rural areas, obtaining adequate access to health information is often difficult. Considering that access is dependent not only on the availability of information, but also on the way it is presented, we based our scenarios on CAD patients’ routine practices and applied plain language and pictorial components, when applicable. We considered the “Understand” skill as a function of cognitive and general literacy skills, as well as previous knowledge and experiences within the disease-management process, and drafted our scenarios and questions accordingly. For the “Evaluation” skill, we prepared two scenarios for each topic, which were similar in content but differed in format/layout, credibility of the sources, and relevance to general population vs. asthma/COPD. The aim was to assess patients’ ability to judge the information in paired scenarios based on their relevance, trustfulness, comprehensiveness, and applicability to their disease management. Although some of the qualities are somewhat subjective, the two scenarios were kept similar, but had some significant differences that allowed the participants to respond to the question. For the “Communication” skill, we focused largely on patient and care provider interactions, and designed our scenarios and questions to assess the patient’s ability to ask necessary questions and express their concerns and opinions. For the “Use” skill, we assessed patients’ abilities and motivation to apply the information in self-management practices. Lastly, for the “Numeracy” skill, we included numbers in both scenarios and questions to assess participants’ abilities to apply numerical information in their decision-making processes.

Patients’ and health professionals’ involvement in the conceptualization of our tool allowed us to confirm that a performance-based HL tool, based on the characteristics mentioned above, is needed. As a secondary benefit, such a tool could help to identify the patient’s HL gaps to facilitate implementation of interventions to address such gaps [73,74,75,76,77]. 

Our study had limitations. The VAHLT was only evaluated with English-speaking patients, and therefore requires validation in other languages. In addition, we recruited participants from a specialty care setting, where patients may have possessed different disease-specific knowledge or perceptions than CAD patients from the general population. To address this issue, we have now collected additional data from patients recruited from the community, and expect to be able to confirm the generalizability of our instrument. Furthermore, we simplified the reading level of the VAHLT to a 5th-grade level to ensure that differences in score were attributable to actual CAD-related functional HL skills and not general literacy, prior disease-related knowledge, or knowledge of medical terminology. However, a reduction of the reading level could affect the assessment of real-world abilities, where confusing or complex terminology/language is commonly seen by patients in health-system encounters [76,77,78,79]. Finally, the length of our measurement tool (nine topics, 44 questions) with an average completion time of approximately 45 min decreased the feasibility for the current version of the tool to be used in clinical practice.

## 5. Conclusions

In this paper, we described the integrated approach taken to develop the VAHLT. We employed a collaborative research and exploratory process aimed at increasing community involvement in our study. A major contribution of this article is that it summarizes the comprehensive process of conceptualizing and developing a performance-based HL measurement tool and highlights the key elements of the development process. We hope our insights will help researchers and clinicians to not only develop measures to address HL gaps in a comprehensive way, but also identify areas in health organizations that must be improved to create HL-competent healthcare systems.

Detailed results on psychometric and clinical validation studies will be addressed in future publications. We also plan to assess the impact of interventions, such as asthma education or pulmonary rehabilitation, on HL scores using VALHT. Enhanced functional measurement of HL will facilitate the development and testing of HL-based interventions to ultimately improve patient outcomes and reduce the burden on the healthcare system.

## Figures and Tables

**Table 1:** The 12 disease-management topics with sample corresponding questions and response options.

Disease-Management Topic	Tool Version (Asthma/COPD)	Sample Question	Response Options
Inhaler Use	Both	Using the information provided in the passage, after putting the metered-dose inhaler (M.D.I.) in your mouth, what is the next step? (Pick only one)	(A) Tilt my head back slightly. (B) Press the canister down as I take a deep breath. (C) Seal my lips around the mouthpiece. (D) Hold my breath for 5 to 10 s.
Prednisone	Both	After using prednisone, you have a headache and feel like you want to throw up. Using the information provided in the passage, what should you do? (Pick only one).	(A) Stop taking prednisone and take an over-the-counter medication for my headache. (B) Call someone I know who also takes prednisone and ask what they would do. (C) Call or visit my doctor and ask what I should do. (D) Stop taking the prednisone and just continue taking my regular inhalers.
Flu (Influenza)	Both	Using the information provided in the passage, which of the following is NOT correct about the flu shot? (*Pick only one*)	(A) It is safe. (B) Its benefits last for several years. (C) It is free for most people in Canada. (D) It saves lives.
Weather and Air Quality	Both	Looking at the weather forecast and air quality health index for each day, which day would be the best day for you to go outside because of the air quality? (*Pick only one*). *Note:* Forecast with index provided.	(A) 31 January (B) 1 February (C) 2 February (D) 4 February
Navigation (Hospital Map)	Both	The lung doctor asks you to do a breathing test at the Lung Function Lab. After doing your breathing test at the Lung Function Lab, you need to pick up your medications at the Pharmacy before going home. Using the Map passage, which elevator would you pass by as you go from the Lung Function Lab to the Pharmacy? (Pick only one).	(A) Elevator 2. (B) Elevator 3. (C) Elevator 4. (D) Elevator 5.
Breathing Control	Both	Using the information provided in the passage, which of the following is the correct method for pursed-lip breathing technique?	(A) Breathe in slowly through your nose and breathe out slowly through your mouth with tight lips. (B) Breathe in through your mouth and slowly breathe out through your nose. (C) Take a long and deep breath in through your nose and breathe out quickly through your mouth with tight lips. (D) Breathe in slowly through your mouth and breathe out slowly through your mouth with tight lips.
Diet and Nutrition	Both	You are invited to a restaurant for a dinner event. You noticed that the entire dinner menu is heavy and greasy. You want to enjoy the dinner with your friends but you also want to make the right decisions considering the tips you received from the dietitian. Using the information provided in the passage, what should you do? (*Pick only one*)	(A) I will eat my meal slowly and with small bites. (B) I will order a green salad appetizer. (C) I will eat a small portion of the food. (D) I will drink plenty of water during my meal to avoid the greasiness of the meal.
Exercise	Both	Using the information provided in the passage, which of the following is true about cardio and strengthening exercises? (*Pick only one*)	(A) Cardio exercise is the only way to prevent heart and lung disease. (B) You should not swim and lift weights on the same day. (C) Both cardio and strengthening exercises improve your health more than if you only perform either one. (D) Strengthening exercises improve heart muscle strength more than cardio exercise.
Asthma Instructions	Asthma	Using the information provided in the passage, when should you arrange to see your doctor? (*Pick only one*)	(A) When my asthma gets worse with exercise. (B) When my medication is not helping. (C) When I have a cough or get wheezy with exercise. (D) As soon as my breathing gets worse.
Peak Flow Meter	Asthma	If your personal baseline average peak flow measurement is 360, Based on the information on the chart, on which day(s) should you have called 911 or gone to emergency room? *Note:* Peak flow measurements provided.	(A) Tuesday. (B) Wednesday. (C) Thursday and Friday. (D) Friday.
Rehab Program for COPD	COPD	Using the information provided in the passage, what is the main goal of a lung rehab program? (*Pick only one*)	(A) Treat a person’s COPD. (B) Help a person to make their muscles stronger. (C) Prevent a person from becoming depressed. (D) Improve a person’s quality of life.
Mucus Control	COPD	Using the information provided in the passage, which of the following is the major difference between huffing and controlled coughing for clearing mucus? (*Pick only one*)	(A) Sit down and relax. (B) Hold breath for 3 s after breathing in. (C) Take a deep breath to fill up your lungs full. (D) Breathe out in 3 short bursts.

**Table 2:** The sample questions for each HL domain measured.

Domain	Sample Question	Response Options
Access	After visiting the lung doctor at the Lung Centre, you need to pick up your medications at the Pharmacy. Using the Map scenario, which of the following 2 places would you pass by if you took the shortest route from the Lung Centre to the Pharmacy? (Pick only one).	(A) Community Care Access Centre and Cardiac Diagnostic Services. (B) Cafeteria and Fracture Clinic. (C) Fracture Clinic and Community Care Access Centre. (D) Cafeteria and Gift Shop.
Communicate	You are talking to a friend who also has asthma. Your friend tells you they stopped taking their medications because they have not had any asthma attacks or trouble breathing recently. Using the information provided in the passage, what would be the best advice to give your friend? (*Pick only one*)	(A) You should call a nurse hotline to consult. (B) You should stop now and continue taking medication again when your symptoms start. (C) You should continue taking the medication, but consult with your doctor. (D) You should take less medication for a while and see how you feel.
Evaluate	Comparing the information from SAMPLE A and SAMPLE B, which sample provides reliable information that you would be willing to apply in your own disease management?	(A) SAMPLE A. (B) SAMPLE B.
Numeracy	If a person uses 4 puffs of their controller inhaler a day, they should refill their inhaler prescription every 30 days. If their doctor tells them to increase the number of inhalations to 6 puffs a day, when would they need to refill their inhaler prescription? (*Pick only one*)	(A) Every 30 days. (B) Every 60 days. (C) Every 15 days. (D) Every 20 days.
Understand	Using the information provided in the passage, what does the term ‘medication interaction’ mean? (Pick only one)	(A) Prednisone works well only when you take all the pills. (B) Prednisone can cause certain side effects for some people. (C) Prednisone must be taken as prescribed by the doctor. (D) Prednisone can change the effect of other medications if taken at the same time.
Use/Apply	You notice your breathing is getting worse. Using the information provided in the passage, what do you need to do? (*Pick only one*)	(A) Continue to only use the controller inhaler. (B) Stop using the controller inhaler and only use the reliever inhaler. (C) Continue to use the controller inhaler and add extra puffs of the reliever inhaler. (D) Stop using all my inhalers and call 9-1-1.

## Data Availability

Data available on request due to privacy restrictions. The data presented in this study are available on request from the corresponding author. The data include interviews with patients and clinicians and are not publicly available due to a lack of explicit agreement from participants to share their data in this way.

## Appendix A. Sample Measurement-Tool Passage

Inhalers and When to Use Them The following information is shared by the pharmacist about the inhalers you have been given. Please read the text below and use the information to answer questions 1 to 9. Feel free to look back to the text to help you answer the questions. There are 3 types of inhalers: - Controller Inhalers - Reliever Inhalers - Combined Controller and Reliever Inhalers Controller Inhalers (Preventer/Maintenance Inhalers) These inhalers need to be used every day, even when a person is breathing well. You should not stop using your controller inhaler without first talking to your doctor. It is the controller inhaler that helps you to breathe well. Reliever Inhalers (Rescue Inhalers) These inhalers are used when needed. For example, if your breathing is getting worse (such as the first signs of a cold, coughing at night, getting wheezy all the time, and/or have a tight chest), the rescue inhaler helps give quick relief. You still need to continue using your controller inhaler in addition to taking extra puffs of your rescue inhaler. Another time the rescue inhaler is used is when you know you get breathing problems from exercising. In this case, you would use the rescue inhaler 30 min before exercising. This usually allows you to breathe more easily while exercising. Combined Controller and Reliever Inhalers Some inhalers work both as a controller inhaler and a rescue inhaler. Your doctor will explain how to use them. Make sure you use your inhalers as instructed by your doctor. If you have any questions about the type of inhalers, talk to your doctor or pharmacist. How to correctly use an inhaler Most people with problems breathing use what is called a ‘metered-dose inhaler’ (or M.D.I.). Here is how to correctly use a metered-dose inhaler: 1. Shake the inhaler for at least 5 s. 2. Remove the cap from the inhaler. 3. Tilt your head back slightly. 4. Breathe out slowly and away from your inhaler. 5. Put the inhaler in your mouth. 6. Seal your lips tightly around the mouthpiece. 7. Press the canister down as you take a slow deep breath. Try to fill your lungs. 8. Take the inhaler mouthpiece out of your mouth. 9. Hold your breath for 5 to 10 s. 10. Breathe out through your nose, if you can. 11. If you need to use a second puff, always wait 30 to 60 s before the second puff. 12. Rinse your mouth or gargle, if using a steroid inhaler.
